# SOCIOECONOMIC DETERMINANTS OF INFORMAL CAREGIVING IN INDIA

**DOI:** 10.1093/geroni/igz038.1811

**Published:** 2019-11-08

**Authors:** Kent Jason G Cheng, Janet M Wilmoth

**Affiliations:** Syracuse University, Syracuse, New York, United States

## Abstract

This study investigates the socio-economic determinants of informal caregiving for elderly parents in urban India, with a focus on caste differences. Bivariate and multivariate logistic regression models of caregiving are estimated with data from 2011 data of Osaka University’s Preference Parameters Study. Three types of caregiving are examined: helping with housework, financial assistance, and providing care. The control variables include: age, sex, marital status, wealth, religiosity, self-rated health, parents requiring care, number of siblings, and number of co-resident children. The bivariate analysis indicates that the highest caste is significantly less likely than the lowest caste to help with housework (OR=.734, SE=.127). In the fully specified models, there is not a significant difference between caste groups in the likelihood of helping with housework or providing financial assistance, but the highest caste is more likely than the lowest caste to provide care (OR=1.443, SE=.309). Being female and married significantly lowers the odds of each type of caregiving. Wealth increases the likelihood of providing help with housework and financial assistance. When both parents require care, children are more likely to provide financial assistance and help with housework, but when one parent requires care, children are more likely to provide care. Overall, sex, marital status, and wealth are the strongest predictors of helping with housework and financial assistance, whereas sex, marital status, and caste are the most important predictors of providing care. The implications of these findings for aging parents and adult children in urban India are discussed.

